# A 12-Year Retrospective Study of Invasive Amoebiasis in Western Sydney: Evidence of Local Acquisition

**DOI:** 10.3390/tropicalmed3030073

**Published:** 2018-06-26

**Authors:** Ana Domazetovska, Rogan Lee, Chandra Adhikari, Matthew Watts, Nicole Gilroy, Damien Stark, Shobini Sivagnanam

**Affiliations:** 1 Department of Infectious Diseases, Blacktown Hospital, Blacktown NSW 2148, Australia; Ana.Domazetovska@health.nsw.gov.au; 2 Department of Infectious Diseases, Westmead Hospital, Westmead NSW 2145, Australia; Matthew.Watts@health.nsw.gov.au (M.W.); Nicky.Gilroy@health.nsw.gov.au (N.G.); 3 Department of Microbiology, Liverpool Hospital, Liverpool NSW 2170, Australia; 4 New South Wales Health Pathology, Institute of Clinical Pathology and Medical Research, Westmead Hospital, Westmead NSW 2145, Australia; Rogan.Lee@health.nsw.gov.au; 5 The University of Sydney Medical School, Westmead Hospital, Westmead NSW 2145, Australia; 6 Department of Anatomical Pathology, Institute of Clinical Pathology and Medical Research, Westmead Hospital, Westmead NSW 2145, Australia; Chandra.Adhikari@health.nsw.gov.au; 7 Department of Microbiology, St. Vincent’s Hospital, Darlinghurst NSW 2010, Australia; damien.stark@svha.org.au

**Keywords:** amoebiasis, *Entamoeba histolytica*, endemic, local acquisition, colitis, liver abscess

## Abstract

In Australia, amoebiasis is thought to occur in travellers, immigrants from endemic areas, and among men who have sex with men. Prevalence of amoebiasis in communities with immigrants from *Entamoeba histolytica*-endemic countries is unknown. The present study is a retrospective case series analysis of patients with laboratory-confirmed amoebiasis from Western Sydney Local Health District, Australia, between years 2005 and 2016. Forty-nine patients with amoebiasis were identified, resulting in an estimated annual incidence of up to 1.1 cases per 100,000 adults. Many were born in Australia (15/47) and India (12/47). Three patients (3/37) had no history of overseas travel, two others had not travelled to an endemic country, and an additional two had a very remote history of overseas travel; one died of fulminant amoebic colitis. Three patients (3/16) were employed in the food industry and one had a history of colonic irrigation in an Australian ‘wellness clinic’. Patients had invasive amoebiasis with either liver abscess (41/48) or colitis (7/48), diagnosed most commonly by serology. Invasive procedures were common, including aspiration of liver abscess (28/41), colonoscopy (11/49), and partial hepatectomy (1/49). Although rare, local acquisition of amoebiasis occurs in Western Sydney and contributes to significant morbidity and hospital admissions.

## 1. Introduction

Amoebiasis, caused by the parasite *Entamoeba histolytica,* has a worldwide distribution, with an estimated 50 million people being infected [1]. With 40,000–100,000 deaths reported yearly, it is the second leading cause of death from parasitic diseases worldwide [2]. High-risk areas include South Asia, Southeast Asia, the Middle East, and South America [3]. *E. histolytica* is acquired through ingestion of food or water contaminated with faecal cysts; sexual transmission has also been reported, particularly via contact with commercial sex workers [4] or in men who have sex with men (MSM), probably via faecal-oral route [5]. Clinical features of amoebiasis include asymptomatic colonisation, which can progress to invasive disease in the form of amoebic colitis or extra-intestinal disease, with the most common manifestation being a liver abscess [6].

In Australia, amoebiasis is thought to be imported by travellers and recent immigrants from high risk areas [7], with an estimated prevalence of 2% in travellers and 1% in immigrants [8]. Locally-acquired disease affects the MSM population [5], and the Indigenous community in northern Australia [9]. As amoebiasis is not a nationally notifiable disease in Australia, the prevalence of amoebiasis, including the prevalence of locally-acquired cases, is unknown. In this study, we aim to determine the epidemiology of amoebiasis in Western Sydney, an area with a high proportion of immigrants from endemic countries [10], in order to raise awareness and improve the morbidity and mortality associated with invasive disease.

## 2. Materials and Methods 

### 2.1. Study Setting

Western Sydney Local Health District (WSLHD) covers four public hospitals that predominantly see adult patients: Westmead, Blacktown, Auburn, and Mount Druitt hospitals. In 2013, the district had an estimated population size of 876,500; 43% of the residents were born overseas and 45% spoke a language other than English at home in 2011 [10].

### 2.2. Study Cohorts

All patients from WSLHD who had a positive amoebic serology, stool microscopy suggestive of *E. histolytica*/*dispar*, and/or a positive *E. histolytica* polymerase chain reaction (PCR) between years 2005 and 2016 were identified using the laboratory information system and were eligible for inclusion in the study. Electronic and paper medical records of eligible patients were retrospectively reviewed to determine demographics, clinical features, and outcome data. Ethics approval was obtained from the WSLHD human research ethics committee.

### 2.3. Diagnostic Methods

#### 2.3.1. Microscopy

Stool microscopy looking specifically for ova, cysts, and parasites (OCP) were performed upon clinicians’ requests. Stool specimens were received either fresh or in sodium acetate-acetic acid-formalin (SAF) fixative. When there was no travel history provided, all unstained stool samples underwent microscopic examination (wet preparation) for leucocytes and erythrocytes and subsequently a lateral flow assay was performed for giardia and cryptosporidium antigens only. Apart from a standard wet mount, the laboratory would prepare and examine stool specimens stained with a modified iron-haematoxylin stain (Para-Stain. Catalogue number MV1284, Oxoid Australia Pty. Ltd., Thebarton, South Australia) when a relevant travel history was provided and/or when a specific request for *E. histolytica* examination was received.

#### 2.3.2. Serology

Patients’ sera were batched and *E. histolytica* serology was performed once per week at the Centre for Infectious Diseases and Microbiology Laboratory Services, Westmead, using an indirect haemagglutination test (IHA) according to the manufacturer’s instructions (Cellognost Amoebiasis, Siemens Healthcare Diagnostics Products GmbH, Marburg, Germany from 2005 to May 2013; ELI.H.A Amoeba, ELITech Microbio, Signes, France from May 2013 to November 2015). The result of the IHA test was reported as a haemagglutination titre. Titres greater than 64 using the Siemens test and greater than 80 using ELITech were regarded as positive.

#### 2.3.3. PCR 

*E. histolytica* PCR was performed at St Vincent’s Hospital, Darlinghurst, upon clinician request. A laboratory-validated in-house PCR targeting the 18S rRNA genes of *E. histolytica* was used to identify *E. histolytica* [11] between 2005 and November 2015. This was subsequently replaced with RT-PCR kit, Easy Screen Enteric Protozoan Detection Kit (Genetic Signatures, Sydney, Australia) [12].

#### 2.3.4. Histopathology

Tissue samples were referred to the Department of Anatomical Pathology, Institute of Clinical Pathology and Medical Research at Westmead Hospital. Colonic biopsies were stained with haematoxylin and eosin; the diagnosis of amoebic colitis was reported when *E. histolytica* trophozoites were seen under light microscopy.

### 2.4. Definitions

Asymptomatic colonisation was defined as the presence of *E. histolytica* in stool in the absence of colitis or extra-intestinal manifestations. Amoebic colitis referred to symptomatic patients with microbiological or histopathological evidence of amoebiasis, without an established alternate cause. Amoebic liver abscess (ALA) was defined as any patient with microbiological or histopathological evidence of amoebiasis with an abscess demonstrated on liver imaging. Patients with positive bacterial cultures of the aspirated liver abscess or blood with borderline positive amoebic serology were excluded from the study.

Invasive procedure was defined as having a colonoscopy, peripheral insertion of central catheter (PICC) for intravenous antibiotics, aspiration of ALA, or having a surgical resection.

## 3. Results

From a total of 173 patients tested by serology, 63 had positive titres and 49 met the inclusion criteria for this study. All 49 cases were positive by serological testing. In addition, 4 of 13 serology-positive cases that had faecal concentrate examination performed were positive for *E. histolytica/dispar*. *E. histolytica* was positive on liver abscess fluid from three patients and faecal samples from two patients when tested by PCR. Interestingly, these five PCR positive cases were not detected by microscopy. Another four patients were positive for amoebae on histopathology of colonic biopsy. These four patients had already been identified as positive by microbiological tests (serology: four; stool microscopy: two). Amongst the excluded cases, 9 had a clear alternate diagnosis, with 8 of these having borderline positive serology. Another four had a history of previous treatment for amoebiasis. One had no record for review.

### 3.1. Demographic Data and Risk Factors (Table 1)

There were more male than female patients; none were HIV positive. Where sexual history was recorded (*n* = 9), all were heterosexuals. Many patients were born in Australia and India. Among the Australian-born patients, 3/15 had never travelled overseas. One was a female hospital volunteer with fulminant amoebic colitis and sepsis. The other two were men with ALA, one of whom had previous colonic irrigation at a ‘wellness clinic’ and at home. Two additional men born in Australia with amoebiasis proven on colonoscopy had only travelled to the USA and Japan. One intermittently lived with a family member who had travelled to Southeast Asia. Another Australian-born male patient with ALA had last travelled to Fiji 30 years prior. One male patient with ALA was born in Malta and had no overseas travel for 46 years since migration. Occupation was recorded for 16 patients, with three cases employed in the food industry (Table 1).

### 3.2. Clinical Features and Investigations (Table 2)

The three main presenting symptoms were fever, right upper quadrant pain, and diarrhoea. There were 41 cases of ALA. Seven had amoebic colitis. The eosinophil count was measured in 48 patients and was elevated in 13, with a range of 0.6–3.2 × 10^9^/L. All ALA was located within the right lobe, with a single lesion, except for two patients who had multiple abscesses within the right lobe. The size of the abscesses varied, with the largest measuring 15 cm in diameter (Table 2).

### 3.3. Management

Of the 49 cases with invasive amoebiasis, four patients were seen through an outpatient clinic and 45 were admitted to hospital (Table 2). There were 23 surgical admissions, of which 17 were managed under the upper gastrointestinal surgical team and six under general surgery. The other 22 cases were medical admissions, of which 17 were managed under gastroenterology and five under infectious diseases teams. Most patients (43/49) received antibiotics (metronidazole) active against *E. histolytica* before a diagnosis was made, often at a dose lower than that recommended for an ALA. One patient with no overseas travel history died of fulminant amoebic colitis prior to receiving appropriate therapy; she was treated empirically with piperacillin/clavulanate and ciprofloxacin instead. This patient had positive *E. histolytica* PCR on stool and a high antibody titre (1280) but died prior to the results being available. Treatment with an intraluminal agent (paromomycin) was documented to be given to 30/49 patients.

Overall, 36/49 patients underwent an invasive procedure, with 28/41 ALA being aspirated. One patient had partial hepatectomy for the liver lesion, with the diagnosis of an ALA subsequently made by serology following outpatient referral to an infectious diseases physician. Of the 13 patients with ALA who did not undergo an invasive procedure, eight were admitted under a medical team, one was managed as an outpatient, and four were admitted under surgery with infectious diseases team consulting. These 13 patients had ALA with a range of sizes, including an abscess of 15 cm diameter with no complications recorded. There was no history of relapsed amoebiasis documented in any of the 49 patients.

## 4. Discussion

**Figure 1:**
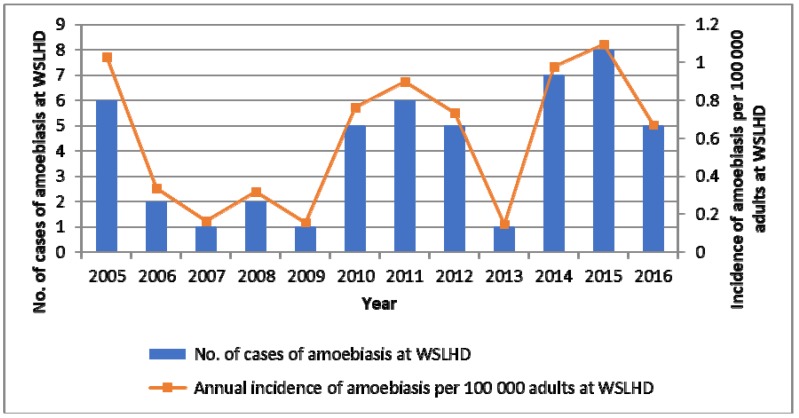
Incidence of invasive amoebiasis at Western Sydney Local Health District (WSLHD) between years 2005 and 2016. Population size estimates in WSLHD per year were derived from the following website: http://www.healthstats.nsw.gov.au/. Patients 15 years and over were considered adults.

This present study is the first epidemiological study looking at invasive amoebiasis in New South Wales, Australia, where *E. histolytica* is not considered to be endemic. We found that although amoebiasis was associated with significant morbidity and mortality, it was a rare infection with an estimated annual incidence of up to 1.1 cases per 100,000 adults in ethnically-diverse Western Sydney (Figure 1). This was likely an underestimate, as it did not capture cases diagnosed in the private sector. The majority of our cases had invasive amoebiasis with a liver abscess. Microscopy is an insensitive test for detection of *E. histolytica* [13], and PCR testing was referred to an external laboratory upon clinician request. As a result, our main selection criterion was positive serology using IHA method. This test is known to have a specificity of over 90% and a high sensitivity for invasive amoebiasis [14], with the most common manifestation being ALA. In addition, only patients with documented travel history had stool concentration (Mini Parasep SF Faecal Parasite Concentrator, Apacor, Workingham, England) and microscopy for *E. histolytica*; this was likely to have had a negative impact on the rate of detection of amoebic colitis. We were also unable to establish the rate of carriage of *E. histolytica* cysts/trophozoites in our patients with ALA, as stool tests were not done in most cases. Finally, as our data largely captured symptomatic patients seen in the hospital setting, there may be a greater burden of asymptomatic colonization in Western Sydney that remains undiagnosed.

With at least three patients with no previous travel history diagnosed with amoebiasis, our study provides evidence that amoebiasis can be acquired locally in Australia. Previous studies have only shown evidence for local acquisition in northern Australia [9,15] and within certain population groups such as MSM [11]. An additional two cases in our study had only travelled to the USA and Japan and two others had a very remote travel history. Although prolonged latency period could not be excluded [16], these cases probably reflect more recent local acquisitions. Hence, amoebiasis should be considered in the differential diagnosis of patients presenting with liver abscesses or colitis, even if there is no travel history.

In our population, there was a high incidence of invasive procedures among patients with amoebiasis, including aspiration of liver abscesses and one case of partial hepatectomy, despite studies showing medical management alone leading to complete recovery [17,18]. One of the factors that contributed to diagnostic delays was a lack of consideration of the diagnosis. Due to the tests being batched, even when the possibility of amoebiasis was raised, there were delays in obtaining results. In some cases, invasive procedures were performed prior to the availability of confirmatory results. Close liaison with the microbiology laboratory may expedite testing and facilitate early diagnosis in such cases. Given that misdiagnosis and delayed diagnosis can lead to fulminant and necrotising colitis [19], and in some cases, death [20], empiric therapy with high-dose metronidazole is of paramount importance in all patients, irrespective of travel history, pending diagnostic test results.

Amoebiasis is not a nationally notifiable disease in Australia. However, our study raises a number of public health concerns. Firstly, three patients were employed in the food industry. Food handlers are well represented in the literature as an at-risk group for transmission of parasitic infections [21,22,23]. For this reason, regular stool screening for parasites in food handlers is practiced in some endemic countries [23,24] and demonstration of stool clearance is required in others [25]. Another public health concern raised in our study was the risk of amoebiasis transmission during colonic irrigation. One patient with amoebiasis had never travelled overseas but had undergone colonic irrigation, a reported source of amoebiasis outbreaks [26]. Public health involvement would help identify and treat potential point source outbreaks from such practices. Finally, one patient with no known history of travel to endemic countries had regular contact with a family member from Southeast Asia. Contact tracing may be necessary to reduce the burden of asymptomatic colonisation, including the spread of infections between household contacts and family members.

Our study has several limitations. This retrospective study has missing data on important risk factors for local acquisition of amoebiasis such as sexual practices and, in some instances, full travel history. We were not able to capture the true prevalence of amoebiasis in Western Sydney, as our data was limited to adult patients reviewed at public hospitals only. Given the small numbers, we were not able to perform meaningful statistical analyses to identify risk factors that may be associated with local acquisition of amoebiasis. Despite these limitations, we believe that our study highlights amoebiasis as a neglected disease of the Western world associated with significant morbidity and mortality and emphasizes the need to have a high index of suspicion even in patients without a travel history.

## 5. Conclusions

Our study suggests that amoebiasis is a significant cause of morbidity and hospital admissions in Western Sydney, perhaps greater than currently recognised and estimated by this study. There is a need for greater awareness and consideration of this diagnosis so that management can be improved and invasive procedures avoided. Diagnosis could also be improved by early testing and result notification in suspected cases. Our findings indicate that amoebiasis acquisition occurs locally in certain Australian communities and future studies that identify local risk factors for transmission are warranted. A prospective multi-centre national surveillance study reporting on demographic and behavioural risk factors, clinical presentation, laboratory diagnosis, and outcomes would better inform public health control and clinical management of amoebiasis in the Australian context.

## Figures and Tables

**Table 1:** Demographic data of patients with invasive amoebiasis at Western Sydney Local Health District between 2005 and 2016.

**Demographics**	No. of Cases *
Male	43/49 (88%)
Age (median; interquartile range)	(49; 34, 61)
**Country of Birth**	
Australia	15/47 (32%)
India	12/47 (26%)
Other ^†^	19/47 (40%)
**Travel History**	
No overseas travel	3/37 (8%)
Remote travel only	2/37 (5%)
Travel to non-endemic countries only	2/37 (5%)
Employed in food industry	3/16 (19%)

* The denominators refer to the number of patients in whom this information was recorded. ^†^ Philippines (*n* = 1), Afghanistan (*n* = 3), England (*n* = 3), Pakistan (*n* = 2), Vietnam (*n* = 1), Poland (*n* = 1), Serbia (*n* = 2), Burma (*n* = 1), Fiji (*n* = 2), China (*n* = 1), Samoa (*n* = 2), Sri Lanka (*n* = 1), Malta (*n* = 1).

**Table 2:** Clinical features, diagnostic testing, management, and outcome data of patients with invasive amoebiasis managed at Western Sydney Local Health District between 2005 and 2016.

**Clinical Features**	**No. of Cases**
Fever	39/49 (80%)
Right upper quadrant pain	35/49 (71%)
Diarrhoea	17/49 (35%)
**Positive Diagnostic Investigations**	
Serology	49/49 (100%)
PCR (liver aspirate)	3/6 (50%)
PCR (Stool)	2/3 (67%)
Stool microscopy	4/13 (31%)
Histopathology	4/11 (36%)
Diagnosis *	
Liver abscess	41/48 (85%)
Amoebic colitis	7/48 (15%)
**Management and Outcomes**	
Admission	
Surgical	23/45 (51%)
Medical	22/45 (49%)
Not admitted	4/49 (8%)
**Treatment**	
Received an antibiotic active against *E. histolytica* prior to diagnosis	43/49 (88%)
Received a luminal agent	30/49 (61%)
Invasive procedure	36/49 (73%)
Aspiration of hepatic abscess	28/41 (68%)
Partial hepatectomy	1/49 (2%)
Colonoscopy	11/49 (22%)
Peripherally inserted central cannula	4/49 (8%)
Death	1/49 (2%)

* Type of amoebiasis could not be determined in one patient due to insufficient information. PCR: polymerase chain reaction.
